# Integrated Decentralized Training for Health Professions Education at the University of KwaZulu-Natal, South Africa: Protocol for the I-DecT Project

**DOI:** 10.2196/resprot.7551

**Published:** 2018-01-25

**Authors:** Pragashnie Govender, Verusia Chetty, Deshini Naidoo, Ntsikelelo Pefile

**Affiliations:** ^1^ College of Health Sciences University of KwaZulu-Natal Durban South Africa

**Keywords:** decentralized clinical training, health science, South Africa, health care

## Abstract

**Background:**

The Integrated Decentralized Training (i-DecT) project was created to address the current need for health care in South Africa among resource poor climates in rural and periurban settings. The University of KwaZulu-Natal (UKZN) in South Africa has embarked on a program within the School of Health Sciences (SHS) to decentralize the clinical learning platform in order to address this disparity. Framed in a pragmatic stance, this proposal is geared towards informing the roll out of decentralized clinical training (DCT) within the province of KwaZulu-Natal. There currently remains uncertainty as to how the implementation of this program will unfold, especially for the diverse SHS, which includes specialities like audiology, dentistry, occupational therapy, optometry, pharmacy, physiotherapy, speech-language pathology, and sport science. Consequently, there is a need to carefully monitor and manage this DCT in order to ensure that the participating students have a positive learning experience and achieve expected academic outcomes, and that the needs of the communities are addressed adequately.

**Objective:**

The study aims to explore the factors that will influence the roll-out of the DCT by developing an inclusive and context-specific model that will adhere to the standards set by the SHS for the DCT program at UKZN.

**Methods:**

Key role players, including but not limited to, the South African Ministry of Health policy makers, clinicians, policy makers at UKZN, clinical educators, academicians, and students of UKZN within the SHS will participate in this project. Once the infrastructural, staffing and pedagogical enablers and challenges are identified, together with a review of existing models of decentralized training, a context-specific model for DCTl will be proposed based on initial pilot data that will be tested within iterative cycles in an Action Learning Action Research (ALAR) process.

**Results:**

The study was designed to fit within the existing structures, and emerging framework and memorandum of understanding between the partners of this initiative, namely, the Ministry of Health and UKZN in order to develop health care professionals that are competent and prepared for the changing dynamics of healthcare in a developing world.

**Conclusions:**

It is envisioned that this study, the first to include a combination of health professionals in a DCT platform at UKZN, will not only contribute to effective service delivery, but may also serve to promote an interprofessional cooperation within the SHS and tertiary institutions in similar settings.

## Introduction

### Background

There has been a global debate on the effectiveness of health care education in regards to preparing graduates for the realities of work [1]. In their landmark paper, Frenk et al (2010) [1] call for instructional changes to facilitate more efficient preparation of health professional graduates to ensure improved health outcomes and service delivery, especially within underserved areas. With this, there is a growing need to review how programs address issues of social accountability, where the responsibility of graduates is to serve as advocates for the marginalized and disenfranchised, particularly in rural areas [2,3,4]. In reviewing this, there is a need to explore the relationships and interactions between clinicians, patients and health services, as well as the university and professional expectations to strengthen various curricula [5]. Concurrent policy changes within the South African Ministry of Health call for a review of the strategies and teaching methods presently utilized to prepare health professional graduates for practice.

Within the South African context, the National Health Act (2003) [6] was pivotal in establishing a decentralized health system in the country. Within the district health system, hospital-based service delivery is offered at quaternary, tertiary and district levels. Community-based services are delivered through community health care centers and primary health care clinics. In the province of KwaZulu-Natal, the high incidence of disease (eg, HIV, tuberculosis, chronic non-communicable diseases, mental health, injury and violence, and maternal and child mortality) has inevitably placed a high burden on the public health system [7,8]. To address this burden of disease, the Negotiated Service Delivery Agreement [9] was designed to promote intersectoral performance around the delivery of identified outputs. For the health sector, this priority meant improving the health status of the entire population, with four main outputs (ie, increasing life expectancy; decreasing maternal and child mortality; combating HIV and acquired immune deficiency syndrome [AIDS], and decreasing the burden of disease like tuberculosis; and strengthening health system effectiveness). The National Health Insurance (NHI) [10] was introduced to redress the disparity between private and public access to health care. With this, the reengineering of primary health care (PHC) served as a concurrent strategy to the NHI. PHC aims to increase accessibility to health services and redress former inequalities [10]. PHC is an approach to health care that includes health promotion, disease and disability prevention, and rehabilitation [10]. This approach stresses the need to shift from a purely curative approach to health care toward encouraging health care practitioners to consider the impact of the social determinants of health. According to NHI policy [10], PHC services should focus on health promotion and prevention while still ensuring quality curative and rehabilitative services.

Despite the introduction of these policies, implementation at the grassroots level remains a problem, with limited literature around current PHC practice being available. Health professionals are challenged to provide services at both district level hospitals and the PHC level [3]. District level hospitals and PHC clinics are predominantly staffed by community service health professions. Community service is the mandatory year-long service that all South African–trained health care practitioners must undertake following their graduation. Within this community service year, health professionals are expected to work within multidisciplinary teams to deliver context-relevant services to marginalized communities in resource constrained settings in rural and peri-urban South African communities. This highlights the need for health professions educators to ensure that graduates are prepared to meet the demands of their entry into the work field, and that the teaching methods presently used are effective in developing the knowledge and skills required for professional practice.

Health education programs use service learning placements as a platform to facilitate transference of theory into practice. The practice of professional knowledge and skills during service learning placements exposes graduates to authentic working environments and equip them with technical competency, critical reasoning, ethical conduct, social attributes, and a sense of social responsibility so that they are able to serve as advocates for their clients [2,11,12,13]. Transformative learning approaches have thus been suggested as an option to promote acquisition of ethical and socially accountable practice. It is postulated that in using critical reflection of students’ experiences, the development of new concepts can be fostered. Moreover, using rational discourse where students’ assumptions are challenged and where they are expected to reason though their decisions, help to promote the development of professional reasoning and practice [14]. Within health education programs, there is growing support for decentralized service learning placements or decentralized clinical training (DCT). Current service placements are predominantly in well-resourced tertiary and district hospitals in urban areas. In contrast, rural DCT placements offer students exposure to the realities of rural practice, different levels of care (eg, PHC clinic and community health care centers) and allows for more active engagement with the community [15,16,17,18,19,20]. Van Schalkwyk et al’s study [16] revealed that medical students found that DCT placements facilitated positive experiences of working at rural district hospitals and with the local community, and allowed the students to gain confidence in their clinical skills and decision-making abilities. Despite support for DCT placements, there is limited South African literature exploring the factors that promote a successful rural placement and positively influence students learning during DCT.

The University of KwaZulu-Natal (UKZN) offers various health science programs which are housed under the College of Health Sciences (CHS). The CHS has initiated steps to ensure that UKZN produces health care professionals who are competent for practice within a PHC model. Additionally, UKZN and the KwaZulu-Natal Department of Health (DoH) have signed a memorandum of understanding (MOU), which will remain operational over the next five years [21]. UKZN’s commitment to the implementation of a decentralized training program for all cadres of health care practitioners is part of this agreement [21]. DCT in primary health care in the context of this study include service placements in DoH PHC sites.

**Figure 1:**
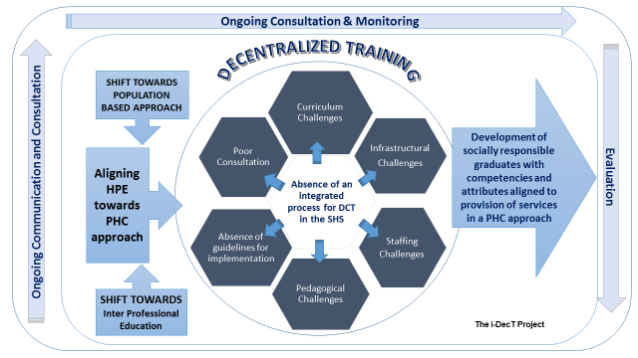
Conceptual framework for the i-DECT Project. HPE: health professions education; PHC: primary health care; DCT: decentralized clinical training; SHS: School of Health Sciences.

It is envisaged that this training would enhance students’ learning through the development of positive attitudes to rural practice and community engagement. Currently, students who are engaged with family medicine and public health modules as part of their medical degree program participate in a decentralized service learning placement. However, this is executed with a small number of students and in one medical discipline. For the School of Health Sciences (SHS), there currently remains uncertainty as to how the implementation of this program will unfold, especially given the diversity of the school, which comprises of eight disciplines (occupational therapy, physiotherapy, pharmacy, optometry, dentistry, biokinetics and sports science, speech and language and audiology). Consequently, there is a need to establish strategies to ensure successful SHS DCT placements, especially exploration into the factors that need to be considered to ensure that students have a positive learning experience. The Integrated Decentralized Training (i-DecT) project has been initiated in response to this need and aims to explore the factors that will influence the roll-out of DCT placements within the SHS at UKZN in order to construct a model of practice for service learning for the school.

### Research Aims and Objectives

The overall purpose of the i-DecT project is to establish an effective, decentralized training program within the SHS at the UKZN in order to:

1. Develop graduates who are socially responsible.
2. Develop graduates who are able to engage in interprofessional practice to improve service delivery in response to the health needs of the country.

In order to ensure that practical solutions are sought, the research questions are exploratory in nature and are described within the specific objectives.

The overall research question (what factors will influence the roll-out of the DCT for the SHS at the UKZN?) is geared toward the development of an inclusive and context-specific model. Figure 1 highlights some of these challenges from a conceptual perspective towards addressing these via the vehicle of DCT. These will be realized by (i) exploring the pedagogical challenges; (ii) the infrastructural challenges; and (iii) the staffing challenges as well as by (iv) identifying principles and factors for inclusion in the model for DCT.

## Methods

The study is located within South Africa in the province of KwaZulu-Natal. Three decentralized clinical training platforms (CTPs) have already been developed towards the optimization of service delivery and inclusion of these sites to become part of the continuous community and clinical placement service and training platforms for the SHS. This study will take on a multidisciplinary approach with the inclusion of eight health disciplines: audiology, dentistry, occupational therapy, optometry, pharmacy, physiotherapy, speech-language pathology, and sport science. These are the disciplines that are currently housed within the SHS, and hence have been included as a homogenous group.

This study will involve the use of multiple methods as an inclusive approach to knowledge generation and legitimization [22] and will provide the opportunity to utilize the strengths of both qualitative and quantitative approaches within this study.

More specifically, an embedded case study design will be used. The SHS is the case under investigation. The study is structured through three phases: a prepilot, a pilot, and a roll-out phase.

### Prepilot Phase

In this phase, an exploration of the pedagogical, infrastructural and staffing challenges will be explored. To ensure methodological diversity, a combination of desk and field research will be used.

#### Desk Research

The desk research component will include the following actions:

- Scoping reviews will be conducted in order to establish existing models of DCT as well as principles to be embedded in interprofessional training of health professionals
- Document reviews will be conducted and will include an analysis of curriculum documents to identify the potential for interprofessional learning and methods of supervision required on site in order to address pedagogical challenges

- Policies, processes and procedures that exist between the academy and the health ministry will be reviewed to ensure effective service delivery and principles to be adhered to
- Infrastructural (ie, space, equipment, and other resources) and staffing requirements (ie, supervisors, clinicians, students) will be assessed for effective service delivery according to norms and standards established for each of the professionals in PHC

#### Field Research

The field research component is outlined as follows:

- Study participants will include clinicians, clinical educators, students, academicians, key stakeholders, and decision makers within the Ministry of Health and the UKZN
- The richness of the data collected will depend on the multitude of perspectives generated from the use of the above sources
- Participants will be recruited based on predetermined selection criteria with purposive sampling techniques
- In order to explore issues of teaching, learning, supervision practices (pedagogy), service delivery through the DCT vehicle of training and human resource and infrastructural challenges, the following methods of data collection [23] will be used:
  - Surveys, focus groups discussions and semistructured interviews with all stakeholders to identify perceptions around DCT and potential challenges, enablers, and opportunities
  - Students will be encouraged to complete blogs as they prepare for the piloting of the DCT
  - Direct observations will occur on site in each of the CTPs to ensure that the infrastructural evaluation is strengthened

### Pilot Phase

In this phase, an exploration of the experiences of students and clinicians will occur within the various CTPs of the DCT program.

#### Action Learning Action Research

Action Learning Action Research (ALAR) can be considered a cyclic process that includes action and critical reflection stages that in turn produce learning. Within the action research cycle in this study, the following processes and methods will be followed:

- Setting of clear goals and preparation for DCT (eg, identification of needs and setting of faculty learning outcomes, developing student learning outcomes, and performance indicators for each outcome)
- Action stage when placement and supervision occurs at the CTP and reflect on experiences with use of blogs and reflective diaries
- Focus group discussions mid-way through placement to identify revisions and changes to be implemented
- A reflective critique following implementation of changes

#### Co-operative Inquiry

A co-operative inquiry with educators and clinicians will include the following stages:

- Innovation/Proposition Stage where evaluation of immediate impact and trial strategies are considered for the DCT model
- Investigative/Action Stage where gathering of baseline data and strategic focus for the DCT at the CTP is determined
- Dissemination/Reaction Stage where information is shared and future foci and strategies are determined that will influence the model for DCT
- Reflection Stage where the intermediate impact is evaluated, followed by further refinement and evaluation in preparation for the roll-out phase

### Roll-Out Phase

The data from the prepilot and pilot phases will be merged in order to establish principles that are essential for an appropriate DCT model based on an interprofessional educational rationale. The model for the DCT program will then be implemented within each of the CTPs.

#### Trustworthiness and Rigor

Techniques for credibility, confirmability, and transferability in this study will include triangulation (methods, source, and analyst triangulation) and member checking / respondent validation in the iterative processes that are inherent in the study. Qualitative and quantitative data will be combined to elucidate complementary aspects of the experiences of the sample in this project. Source triangulation will be ensured by the inclusion of different samples of clinicians, clinical educators, and students. Analyst triangulation will be ensured in the appraisal of the literature and in coding and analysis processes of the qualitative aspects of the research. Reflexive triangulation will also be adopted where reactions and responses of the participants will be recorded. Techniques for confirmability or objectivity will include debriefing, documentation of an audit trail, and reflexivity. Continuous open dialogue with critical readers and mentor will occur [24] in addition to peer debriefing. The researchers in this study occupy an emic (insider) perspective and hence reflexivity and positionality is essential and bracketing of biases may be necessary.

#### Data Analysis

Semistructured interviews and focus group discussions will be audio-recorded. Transcriptions, together with the reflective blogs and diaries, will be exposed to content analysis through the use of computer-assisted qualitative data analysis software (eg, NVIVO, version 11). Both inductive and deductive reasoning will be used to analyze the data on three levels (codes or nodes, categories and themes). Some of the more specific analytical aspects, namely, constant comparisons between groups, use of the group dynamics as a resource, and use of participants as co-analysts [25,26] will also be considered. The surveys selected for various phases of this project will be analyzed descriptively used MS Excel 10 and SPSS version 24. Data from the qualitative and quantitative phases will be merged and integrated to form conclusions and make relevant assertions.

### Ethical Considerations and Boundaries

Given the emergent nature of this study and the absence of a framework for research within decentralized training programs, the researchers anticipate that there may be power dynamics that will have to be negotiated. It is for this reason that the researchers have attended relevant meetings and have been transparent in the processes that are documented within this project protocol. Moreover, key role players have been approached to form part of the research team in order to contribute to the development of a framework for research in this area. Continued consultation and open communication is identified as necessary towards the ethical principle of beneficence in this study. Gatekeeper permissions have been obtained in addition to approval from a research ethics committee (HSS/0727/017). Written informed consent will be obtained from participants prior to initiating the study. Principles of autonomy and anonymity and the right to withdraw will be observed.

## Results

The project was funded in 2016 with ethical clearance approval granted in 2017. The study is ongoing and researchers are in the process of recruiting postgraduate research fellows as study collaborators as well as reviewing contemporary literature. Currently, the project is in the prepilot phase with data collection underway, and the first results are expected to be submitted for publication in 2018.

## Discussion

The changing landscape of health education and requirements of graduates are the driving force behind the current reengineering of the CHS curriculum towards DCT. This new curriculum aims to ensure that the UKZN produces health care professionals who are competent and prepared for the changing dynamics of health care in a developing world. It also aims to guide the acquisition of graduate competency and proposes that health professionals should demonstrate mastery and/or acquire skills in seven key roles, namely, as practitioner, communicator, collaborator, leader, scholar, health advocate, and professional [27].

Currently, most of the disciplines in the school place students in well-resourced hospitals and disadvantaged communities in urban settings for service learning. The concern is that these placements do not adequately equip students with the ability to assess and treat patients in resource-constrained environments or understand how the DoH system works, including the referral pathways. Additionally, students do not have sufficient opportunity to engage with or consult the community or have the opportunity to deliver programs that address health promotion, and primary, secondary and tertiary prevention of disease and disability. Another criticism is that the current service learning placements in the province are fragmented. Therefore, health professions educators are finding it difficult to create opportunities for interprofessional practice. Anecdotal reports indicate that this results in a lack of understanding of the scope of practice between the various health care practitioners, and decreased intrateam communication and planning, which leads to less favorable outcomes for patients.

In taking this project forward, decentralized service placements can offer a solution where students are exposed to and are allowed active engagement with the communities they serve. Furthermore, the MOU between UKZN and KwaZulu-Natal DoH requires the implementation of DCT in KwaZulu-Natal for the next five years. Although this understanding exists between the two parties, there are currently no formal guidelines or processes in place to guide implementation for the SHS at UKZN. Moreover, the absence of baseline information poses a threat to the successful rollout of this program. For a decentralized learning platform to provide a positive learning experience for students, it needs to be careful planned, monitored and evaluated. However, at present there are many uncertainties that require further exploration to ensure a successful implementation.

## Abbreviations

AIDS: acquired immune deficiency syndrome
ALAR: Action Learning Action Research
CHS: College of Health Sciences
CTP: clinical training platform
DCT: decentralized clinical training
DoH: KwaZulu-Natal Department of Health
i-DecT: integrated decentralized training
HPE: health professions education
MOU: memorandum of understanding
NHI: National Health Insurance
PHC: primary health care
SHS: School of Health Sciences at the University of KwaZulu-Natal
UKZN: University of KwaZulu-Natal
